# HIV disclosure and depressive symptoms among pregnant women living with HIV: a cross‐sectional study in the Democratic Republic of Congo

**DOI:** 10.1002/jia2.25865

**Published:** 2022-02-07

**Authors:** Natalia Zotova, Itziar Familiar, Bienvenu Kawende, Fidele Lumande Kasindi, Noro Ravelomanana, Angela M. Parcesepe, Adebola Adedimeji, Kathryn E. Lancaster, Didine Kaba, Pélagie Babakazo, Marcel Yotebieng

**Affiliations:** ^1^ Division of General Internal Medicine Department of Medicine Albert Einstein College of Medicine Bronx New York USA; ^2^ Department of Psychiatry Michigan State University East Lansing Michigan USA; ^3^ School of Public Health University of Kinshasa Kinshasa, Democratic Republic of Congo; ^4^ Department of Maternal and Child Health University of North Carolina at Chapel Hill Chapel Hill North Carolina USA; ^5^ Department of Epidemiology and Population Health Albert Einstein College of Medicine Bronx New York USA; ^6^ Division of Epidemiology, College of Public Health Ohio State University Columbus Ohio USA

**Keywords:** depression, pregnant women, HIV, status disclosure, intimate partner violence, DR Congo

## Abstract

**Introduction:**

Disclosure of one's HIV status may decrease depression and improve the quality of life among people living with HIV. However, there is mixed evidence on the impact of disclosure to partners for pregnant women living with HIV (WLHIV) in areas of intersecting social concerns over disclosure and high prevalence of intimate partner violence (IPV). We assessed the association between HIV disclosure and depressive symptoms among pregnant WLHIV in the Democratic Republic of Congo (DRC) and examined whether the knowledge of partner's status or recent IPV modified this association.

**Methods:**

We utilized data from participants enrolled in a trial to evaluate the effect of continuous quality interventions on long‐term therapy outcomes among HIV‐positive pregnant and breastfeeding women in DRC (NCT03048669). Only pregnant women (*n* = 1392) were included in this cross‐sectional analysis. Between November 2016 and June 2019, enrolled participants completed a survey that included the Patient Health Questionnaire‐9 (PHQ‐9) to screen recent depressive symptoms, questions about disclosure, knowledge of partner's status and IPV. We used linear models to calculate crude and adjusted mean differences (MDs) between disclosure and depressive symptoms. All analyses were stratified by timing of HIV diagnosis.

**Results:**

Disclosure was higher among participants diagnosed prior to current pregnancy (41% to their partners and 24% to family, friends or others) relative to those diagnosed during current pregnancy (21% to partners and 12% to family). About one‐quarter of women reported any type of IPV in the past 12 months. Disclosure to a partner was associated with lower depressive symptoms among women diagnosed prior to current pregnancy (MD −0.55; 95% CI: −1.06, −0.04) but the opposite was observed among those diagnosed during current pregnancy (MD 0.5; 95% CI: −0.4, 1.4). Adjustment for IPV, knowledge of partner's status, age, number of living children and primigravidae did not change MDs substantially.

**Conclusions:**

Women in our sample mostly disclosed to partners despite high IPV burden. The observed association between disclosure to partners and lower depressive symptoms among women diagnosed prior to current pregnancy is consistent with cross‐national evidence. A prospective study among pregnant WLHIV is needed to examine longitudinal effects of HIV status disclosure.

## 1 INTRODUCTION

Depressive symptoms are highly prevalent among pregnant women living with HIV (WLHIV) [1, 2]. A systematic review of studies examining prenatal depression in African WLHIV found that weighted mean prevalence of antenatal depression was 23.4% [3]. Depression negatively affects the quality of life among people living with HIV (PWLH) and compromises the HIV care continuum and health outcomes, including testing, adherence to treatment, viral suppression and mortality [1, 4–8]. Specifically, among pregnant WLHIV, depression has been associated with suboptimal adherence to antiretroviral therapy (ART) [9, 10].

Disclosure of one's HIV status is thought to be beneficial for psychological wellbeing and is encouraged in counselling services for PLHIV [11, 12]. However, there is mixed evidence on the association between disclosure and depressive symptoms particularly from sub‐Saharan Africa [12]. Depressive symptoms’ trajectories also depend on the timing of HIV diagnosis. For example, in a study from Uganda, participants diagnosed with HIV had high depressive symptoms following diagnosis, but their symptoms decreased significantly over time. Disclosure to a partner following HIV diagnosis was also associated with lower depression and positive coping compared with those who did not disclose by the end of 28‐day, 3‐ and 6‐month period [13]. A study in Western Kenya found that although most women (88%) reported positive reactions from their partners upon disclosure, others reported their partners were confused, annoyed or had threatened to leave [14]. Qualitative studies from Malawi and Tanzania found that pregnant WLHIV reported intimate partner violence (IPV), abandonment or divorce following disclosure [15, 16]. Quantitative studies from Tanzania and Zimbabwe have also showed a positive association between experiences of IPV and depression among pregnant WLHIV [17, 18].

On the other hand, nondisclosure, particularly to male partners, has been shown to be an important source of stress in newly diagnosed pregnant WLHIV initiating ART [19]. In qualitative interviews with women in South Africa, women who had not disclosed reported finding it relatively easy to justify to partners the use of medications and frequent visits to a health clinic during pregnancy, but worried about the unsuitableness of these excuses after giving birth [19]. For people in stable partnerships, disclosure of one's HIV status and knowledge of a permanent partner's status can be critical for maintaining their relationship and staying in care, particularly for serodiscordant couples who can benefit from the use of pre‐exposure prophylaxis (PrEP) [20, 21]. A qualitative study among serodiscordant couples in Mozambique found that participants had a strong desire to stay in the discordant relationship and emphasized the need to work together and support each other to ensure PrEP and ART adherence [22].

The effect of HIV status disclosure on depressive symptoms among WLHIV warrants attention especially considering the potential benefits of knowing the partner's status and disclosing one's status. However, the relationship between disclosure, depressive symptoms and IPV, and how knowledge of partner's HIV status can modify this relationship has not been studied among pregnant WLHIV in sub‐Saharan Africa. This study bridges this gap by assessing (1) the association of HIV disclosure with depressive symptoms, and (2) whether knowledge of partner's HIV status or recent experience of IPV (in the past 12 months) modifies this association.

## 2 METHODS

### 2.1 Study design, population and setting

This is a cross‐sectional analysis of enrolment data from a cohort of 1392 pregnant women who participated in a cluster randomized trial evaluating the effect of continuous quality interventions (CQI) on long‐term therapy outcomes among HIV‐infected pregnant and breastfeeding women in Kinshasa, DR Congo (trial registration: NCT03048669).

Between November 2016 and June 2019, all pregnant and breastfeeding women (≤1 year post‐delivery) with positive HIV status who presented for antenatal care, delivery or child health visits in any of the three busiest mother–child health clinics (MCH), in each of the 35 health zones [operational units for health services delivery in the Democratic Republic of Congo (DRC)] in the Kinshasa province were eligible for the parent study [23]. Eligible potential participants were referred to study staff by care providers for consent and enrolment. All enrolled participants provided written informed consent.

**Figure 1:**
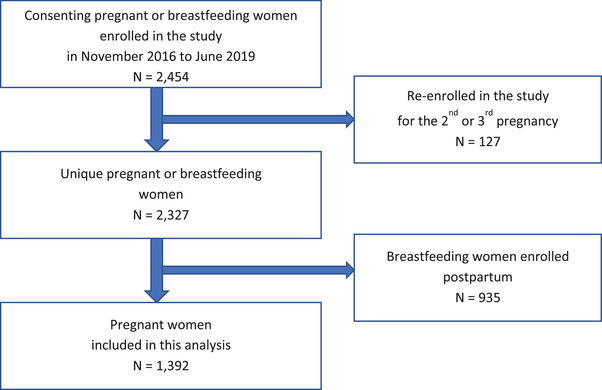
Participants’ inclusion flowchart.

DRC has adopted policies to provide ART to all pregnant and breastfeeding WLHIV regardless of CD4 count or clinical stage (“Option B+”) since 2013. Accordingly, all enrolled participants were on ART. This analysis is limited to women who were enrolled during pregnancy (Figure 1). The study was approved by the Ethical Review Committee of University of Kinshasa School of Public Health and the Ohio State University Institutional Review Board.

### 2.2 Data collection variable definitions

Following consent, enrolled participants were interviewed by trained study nurses (male and female) using a structured questionnaire to collect information on socio‐demographic characteristic, HIV disclosure, knowledge of partner's HIV status, depressive symptoms and history of IPV. Interviews were conducted in a language of respondent's choice (Lingala or French) in a private room at participating MCH clinics and took 40–60 minutes. From participants who agreed to provide a blood specimen, five 50 ml‐spots of blood were obtained via a finger prick on Whatman paper and then used for viral load testing.

The primary outcome of interest was depressive symptoms, screened with the PHQ‐9, a brief instrument based on the nine diagnostic criteria of the DSM‐IV for major depressive disorder [24]. The PHQ‐9 total score ranges from 0 to 27 with five severity categories: minimal (0–4), mild (5–9), moderate (10–14), moderately severe (15–19) and severe (20–27). The country research team in our previous study in DRC translated the PHQ‐9 from French to Lingala, back‐translated from Lingala to French and field tested the Lingala version [25].

HIV status disclosure (undisclosed, disclosed to family/friends/others or disclosed to husband/permanent partner) was the primary exposure. Disclosure was assessed with one question: “Have you told anyone you have HIV?” If women answered yes, they were asked to whom they disclosed. The options included “partner/father of my baby,” “mother,” “sister,” and “other.” Knowledge of permanent partner's HIV status (positive, negative and unknown) was assessed by asking: “Do you know the HIV status of your partner?” If women answered yes, they were asked about their partner's status (positive vs. negative).

Similar to our previous study in the DRC [26], IPV was assessed using questions adapted from items developed by the international WHO Multi‐country Study on Women's Health and Domestic Violence against Women [27], Abuse Assessment Scale (AAS) [28] and the Youth Behavior Risk Survey [29], for an assessment of psychological, physical and sexual violence, respectively. Psychological violence was assessed using the question, “Have you ever been insulted, humiliated, or made to feel afraid by an intimate partner?” Physical violence was assessed using the question “Have you ever been hit, punched, kicked, slapped, choked, or otherwise physically hurt by an intimate partner?” Sexual violence was assessed using the question “Have you ever been forced to have sex or do something sexual you didn't want to do?” If women answered yes, they were asked who perpetrated the violence (“husband/ex‐husband,” “partner/ex‐partner,” “other,” “don't know,” and “refused to answer”) and “did this happen in the past 12 months?” Participants who answered “yes” to any type of violence and “yes” to a question if that happened in the past 12 months were categorized as “yes” for recent IPV and otherwise as “no.”

Based on previous studies among WLHIV in sub‐Saharan Africa [25, 30], potential confounders considered included age, marital status (married/cohabiting vs. other), gestational age, primigravida (yes vs. no), number of living children, time of the HIV diagnosis (prior vs. during this pregnancy), socio‐economic status (SES) and viral load. SES was derived from principal component analysis (PCA) of the following factors: education, average number of household members per room, number of sleeping beds in the household, water source (private pipe vs. other), electricity in the household, cooking fuel type (electrical stove or gas vs. wood/charcoal) and ownership of household goods (mobile phone, radio, fridge, vehicle, bike, motorcycle and car) [23, 31]. We then used the first PCA component to categorize SES into quartiles: 1 (lowest SES), 2, 3 and 4 (highest SES). Viremia measurements were categorized into two groups: unsuppressed (≥1000 copies/ml) and suppressed (<1000 copies/ml) viral load.

### 2.3 Statistical analyses

Demographic and clinical characteristics of participants were summarized with proportions. A standard PHQ‐9 cut‐off score of 10 is commonly used to differentiate participants with depressive symptoms from those without, but studies among PLHIV in sub‐Saharan Africa have yielded mixed results on its sensitivity and specificity [32, 33, 34]. These considerations guided our decision to treat the PHQ‐9 score as a continuous variable and to calculate mean differences (MDs).

Mean PHQ‐9 score was used in models overall and stratified by (1) HIV disclosure; (2) partner's HIV status; and (3) any IPV in the past 12 months. Bivariate and multivariate generalized linear modelling (GLM) were used to calculate the crude and adjusted MDs and the corresponding 95% confidence intervals (CIs) comparing the level of depressive symptoms across levels of HIV disclosure. Because persons newly diagnosed with HIV have been shown to have elevated risks of depression [35] and because they may not have enough time to disclose their HIV status, all analyses were a priori stratified by whether HIV was diagnosed prior to or during the current pregnancy.

To assess whether the association between HIV disclosure and depressive symptoms might be modified by (1) partner's HIV status or (2) recent IPV, separate GLM models were built with each potential effect modifier and their interaction term with HIV disclosure. The significance of the interaction term was assessed at alpha = 0.2. If the interaction term was not significant, the variable was subsequently treated as a potential confounder. All potential confounders found in bivariate analysis to be associated with depressive symptoms (*p*<0.2) were included in multivariate models. Results were also stratified by each potential effect modifier with a statistically significant interaction term.

All statistical analyses were conducted using Stata Version 16.0 [36].

## 3 RESULTS

Out of 2327 participants, 1392 were pregnant at the time of enrolment and were included in this analysis (Figure 1). Most women (55%, *n* = 762) were 24–35 years old (Table 1). The majority of participants (92%, *n* = 1284) were married or cohabiting, and had been pregnant before (non‐primigravida) (88%, *n* = 1231). About two‐thirds of women (63%, *n* = 870) had two and more living children. PCA of factors indicative of participants’ SES revealed that the first component explained 22.3% of variance. It was used as measure of SES to categorize into quartiles. One‐quarter of women (*n* = 344) had the lowest SES, 22.6% (*n* = 308) were in the second quartile, 38.4% (*n* = 523) and 13.7% (*n* = 186), respectively, were in the third and fourth (highest) SES quartiles.

**Table 1:** Socio‐demographic and clinical characteristics of 1392 pregnant women living with HIV from 105 clinics in Kinshasa, the Democratic Republic of Congo

	*N* (%)^a^
Age	
< = 24	227 (16.3)
24–34	762 (54.8)
35+	402 (28.9)
Number of living children	
0–1	522 (37.5)
2	287 (20.6)
3+	583 (41.9)
SES in quartile	
1 (Lowest)	344 (25.3)
2	308 (22.6)
3	523 (38.4)
4	186 (13.7)
Time of HIV diagnosis	
Prior to current pregnancy	783 (56.3)
During current pregnancy	607 (43.7)
Marital status	
Divorced/separated/widowed/never married	106 (7.6)
Married/cohabiting	1284 (92.4)
Gestational age	
First trimester (weeks 1–12)	73 (5.3)
Second trimester (weeks 13–26)	733 (53.5)
Third trimester (weeks 27+)	565 (41.2)
Primigravida	
Yes	161 (11.6)
No	1231 (88.4)
HIV disclosure	
Undisclosed	681 (48.9)
Disclosed to family/friends/others	264 (19.0)
Disclosed to husband/partner	447 (32.1)
Partner's HIV status	
Unknown	775 (55.7)
Negative	375 (27.0)
Positive	241 (17.3)
Any IPV in the past 12 months	
No	909 (75.4)
Yes	296 (24.6)
Psychological violence in the past 12 months	
No	1154 (88.4)
Yes	152 (11.6)
Physical violence in the past 12 months	
No	1166 (89.7)
Yes	134 (10.3)
Sexual violence in the past 12 months	
No	1192 (89.6)
Yes	138 (10.4)
Viral load	
Unsuppressed (≥1000 copies/ml)	441 (33.84)
Suppressed (<1000 copies/ml)	862 (66.16)
Mean PHQ‐9 score (SD)	2.6 (3.8)
Depressive symptoms severity by the PHQ‐9 score	
No depression (0–4)	1081 (77.7)
Mild depression (5–9)	233 (16.8)
Moderate depression (10–14)	50 (3.6)
Moderately severe depression (15–19)	21 (1.5)
Severe depression (20–27)	6 (0.4)

Abbreviations: IPV, intimate partner violence; SES, socio‐economic status.

^a^ Frequencies might not add up to the total for the category because of missing data.

Over half of pregnant WLHIV (51.1%, *n* = 711) had disclosed their HIV status to a partner, family, friends or others. Of these, almost two‐thirds had disclosed to partners (62.9%, *n* = 447). Over half of women (55.7%, *n* = 557) did not know their partners’ status. Among those who knew partners’ status (*n* = 616), 39% (*n* = 241) reported that their husbands/partners were living with HIV. Two‐thirds of participants (*n* = 862) had suppressed viral load. Mean PHQ‐9 score among all participants was 2.6 (SD 3.8). Based on the PHQ‐9 scores, depressive symptoms were minimal in most women (77.7%, *n* = 1081); mild in 16.8% (*n* = 233), moderate in 3.6% (*n* = 50), moderately severe in 1.5% (*n* = 21) and severe in 0.4% (*n* = 6).

Over half of participants (56%, *n* = 783) learned about their HIV status prior to their current pregnancy. Of these, 41% of women (*n* = 319) had disclosed their HIV status to partners, 24% (*n* = 192) to family, friends or others and 35% (*n* = 273) had not disclosed. Forty‐one percent (*n* = 323) of women did not know partner's status. Twenty‐four percent of women reported (*n* = 162) experiencing IPV in the last 12 months. In this group, 74% (*n* = 543) had suppressed viral load. Mean PHQ‐9 score was 1.8 (SD 3.2).

Of the 607 women diagnosed with HIV during current pregnancy, only 21% (*n* = 127) had disclosed their HIV status to partners, 12% (*n* = 72) to family, friends or others and 67% (*n* = 408) had not disclosed. Most women (74%, *n* = 452) did not know partner's status. Twenty‐five percent (*n* = 134) of women experienced IPV in the last 12 months. Fifty‐six percent (*n* = 316) had suppressed viral load. Mean PHQ‐9 score of women in this group was 3.6 (SD 4.3).

In bivariate analyses of data from women diagnosed with HIV prior to this pregnancy, mean PHQ‐9 score was lower among women who had disclosed their HIV status to partners (1.5 vs. 2.1; MD −0.55; 95% CI −1.06, −0.04; *p* = 0.03) or disclosed to family, friends or others (1.9 vs. 2.1; MD −0.18; 95% CI −0.8, 0.45; *p* = 0.58) compared to those who had not disclosed (Table 2). Women who reported that their partners were living with HIV had lower PHQ‐9 scores compared to women who did not know their partner's status (1.4 vs. 2.0; MD −0.63, 95% CI −1.12, −0.13; *p* = 0.01). Mean PHQ‐9 score among women whose partners were HIV uninfected was similar to those who did not know their partner's status (1.8 vs. 2.0; MD −0.22, 95% CI −0.76, 0.32; *p* = 0.42). Mean PHQ‐9 scores were higher among women who experienced any type of IPV in the past 12 months compared to women who had not experienced IPV (2.8 vs. 1.4; MD 1.37; 95% CI 0.77, 1.96; *p*<0.01). Women who had suppressed viral load had similar PHQ‐9 scores compared to women who had unsuppressed viral load (1.7 vs. 2.0; MD −0.27; 95% CI −0.76, 0.22; *p* = 0.28).

**Table 2:** Bivariate associations between HIV status disclosure, partner's HIV status, IPV in the past 12 months, socio‐demographic and clinical characteristics, and depressive symptoms, generalized linear models stratified by the time of HIV diagnosis

	HIV diagnosis prior to current pregnancy				HIV diagnosis during current pregnancy			
	*N* (783)	Mean PHQ‐9 score (SD)	MD (95% CI)	*p*‐value	*N* (607)	Mean PHQ‐9 score (SD)	MD (95% CI)	*p*‐value
HIV disclosure								
Undisclosed (ref.)	273	2.1 (3.6)			408	3.6 (4.4)		
Disclosed to family/friends/others	192	1.9 (3.3)	−0.18 (−0.8, 0.45)	0.58	72	3.1 (3.4)	−0.5 (−1.39, 0.4)	0.28
Disclosed to husband/partner	319	1.5 (2.6)	−0.55 (−1.06, −0.04)	0.03**	127	4.0 (4.6)	0.5 (−0.4, 1.4)	0.28
Partner's HIV status								
Unknown (ref.)	323	2.0 (3.4)			452	3.4 (4.1)		
Negative	272	1.8 (3.3)	−0.22 (−0.76, 0.32)	0.42	103	4.2 (5.5)	0.76 (−0.36, 1.87)	0.18
Positive	188	1.4 (2.3)	−0.63 (−1.12, −0.13)	0.01**	52	3.7 (3.8)	0.25 (−0.85, 1.35)	0.66
Any IPV in the past 12 months								
No (ref.)	515	1.4 (2.9)			393	3.3 (4.1)		
Yes	162	2.8 (3.5)	1.37 (0.77, 1.96)	<0.01***	134	3.6 (3.9)	0.29 (−0.47, 1.06)	0.45
Age								
< = 24 (ref.)	74	1.4 (2.9)			153	4.5 (5.7)		
24–35	432	2.1 (3.4)	0.69 (−0.04, 1.41)	0.06*	329	3.3 (3.8)	−1.17 (2.16, −0.18)	0.02**
35+	277	1.6 (2.8)	0.21 (−0.52, 0.94)	0.57	125	3.3 (3.6)	−1.2 (−2.3, −0.1)	0.03**
Number of living children								
0–1 (ref.)	260	2.2 (3.8)			262	3.8 (4.6)		
2	182	1.6 (2.7)	−0.6 (−1.21, 0.0)	0.05*	105	3.7 (4.3)	−0.12 (−1.1, 0.86)	0.81
3+	342	1.6 (2.8)	−0.6 (−1.15, −0.06)	0.03**	240	3.4 (4.1)	−0.41 (−1.17, 0.35)	0.29
Primigravida								
Yes (ref.)	48	0.7 (1.7)			113	3.7 (4.9)		
No	737	1.9 (3.2)	1.2 (0.68, 1.73)	<0.01***	494	3.6 (4.2)	−0.12 (−1.09, 0.86)	0.82
SES in quartile								
1 (Lowest) (ref.)	187	2.0 (3.1)			157	4.0 (4.6)		
2	178	1.7 (3.5)	−0.3 (−0.97, 0.38)	0.39	130	3.5 (4.1)	−0.48 (−1.49, 0.53)	0.35
3	290	1.5 (2.9)	−0.54 (−1.09, 0.02)	0.06*	232	3.4 (4.4)	−0.58 (−1.5, 0.34)	0.22
4	111	2.0 (3.0)	−0.06 (−0.77, 0.64)	0.86	75	3.2 (4.0)	−0.81 (−1.96, 0.33)	0.17
Marital status								
Divorced/separated/widowed/never married (ref.)	50	2.3 (3.5)			56	3.4 (3.7)		
Married/cohabiting	732	1.8 (3.1)	−0.51 (−1.49, 0.47)	0.31	551	3.6 (4.4)	0.22 (−0.8, 1.25)	0.67
Gestational age								
First trimester (weeks 1–12) (ref.)	54	2.0 (3.2)			19	2.4 (4.4)		
Second trimester (weeks 13–26)	410	1.7 (2.9)	−0.33 (−1.23, 0.56)	0.47	323	4.0 (4.2)	1.54 (−0.45, 3.53)	0.13
Third trimester (weeks 27+)	308	2.0 (3.4)	0.03 (−0.91, 0.96)	0.96	257	3.2 (4.5)	0.79 (−1.22, 2.81)	0.44
Viral load								
Unsuppressed (≥1000 copies/ml) (ref.)	192	2.0 (2.9)			249	3.8 (4.6)		
Suppressed (<1000 copies/ml)	543	1.7 (3.7)	−0.27 (−0.76, 0.22)	0.28	316	3.5 (4.2)	−0.33 (−1.06, 0.4)	0.38

Abbreviations: IPV, intimate partner violence; MD, mean difference; PHQ‐9, Patient Health Questionnaire; SES, socio‐economic status.

Significance levels: ^***^ *p*<0.01; ^**^ *p*<0.05; ^*^ *p*<0.1.

Among women who were diagnosed with HIV during the current pregnancy, mean PHQ‐9 score was higher among those who had disclosed their HIV status to their partner (4.0 vs. 3.6; MD 0.5; 95% CI −0.4, 1.4; *p* = 0.28) and lower among those who disclosed to a family/friend/others (3.1 vs. 3.6; MD −0.5; 95% CI −1.39, 0.4; *p* = 0.28) compared to those who had not disclosed, but the difference was not statistically significant. Women who knew that their partners were living with HIV had similar mean PHQ‐9 scores compared to those whose partners’ status was unknown (3.7 vs. 3.4; MD 0.25, 95% CI −0.85, 1.35; *p* = 0.66). Women who reported that their partners were HIV uninfected had higher mean PHQ‐9 scores compared to women who did not know their partner's status (4.2 vs. 3.4; MD 0.76, 95% CI −0.36, 1.87; *p* = 0.18). Mean PHQ‐9 score was about similar among women who had experienced recent IPV compared to those who had not (3.6 vs. 3.3; MD 0.29, 95% CI −0.47, 1.06; *p* = 0.45). Mean PHQ‐9 scores did not differ significantly among women who had suppressed and unsuppressed viral load (3.5 vs. 3.8; MD −0.33; 95% CI −1.06, 0.4; *p* = 0.38).

In multivariate analyses, the interaction terms between disclosure, partner HIV status or IPV were not statistically significant among participants who were diagnosed prior to their current pregnancy. The strength of association between PHQ‐9 mean score and disclosure of HIV status did not change significantly among women diagnosed prior to current pregnancy [to a partner (MD −0.47; 95% CI −1.14, 0.21; *p* = 0.18) or to family/friend (MD −0.11; 95% CI −0.8, 0.58; *p* = 0.75)] or in the current pregnancy [to partner was (MD 0.43; 95% CI −0.78, 1.64; *p* = 0.48) or to family/friend (MD −0.47; 95% CI −1.38, 0.43; *p* = 0.31)] after adjusting for covariates (Table 3).

**Table 3:** Multivariate associations between HIV status disclosure, partner's HIV status, IPV in the past 12 months, socio‐demographic and clinical characteristics, and depressive symptoms, generalized linear models stratified by the time of HIV diagnosis

	HIV diagnosis prior to current pregnancy		HIV diagnosis during current pregnancy	
	aMD (95% CI)	*p*‐value	aMD (95% CI)	*p*‐value
HIV disclosure				
Undisclosed (ref.)				
Disclosed to family/friends/others	−0.11 (−0.8, 0.58)	0.75	−0.47 (−1.38, 0.43)	0.31
Disclosed to husband/partner	−0.47 (−1.14, 0.21)	0.18	0.43 (−0.78, 1.64)	0.48
Partner's HIV status				
Unknown (ref.)				
Negative	−0.1 (−0.74, 0.55)	0.76	0.5 (−0.76, 1.76)	0.44
Positive	−0.23 (−0.93, 0.46)	0.51	−0.18 (−1.6, 1.24)	0.8
Any IPV in the past 12 months				
No (ref.)				
Yes	1.27 (0.68, 1.86)	<0.01***		
Age				
< = 24 (ref.)				
24–35	0.52 (−0.29, 1.33)	0.21	−1.16 (−2.14, −0.18)	0.02**
35+	0.09 (−0.72, 0.89)	0.83	−1.2 (−2.3, −0.1)	0.03**
Number of living children				
0–1 (ref.)				
2	−0.59 (−1.21, 0.04)	0.07*		
3+	−0.29 (−0.87, 0.3)	0.34		
Primigravida				
Yes (ref.)				
No	1 (0.31, 1.68)	<0.01***		

Abbreviations: aMD, adjusted mean difference; IPV, intimate partner violence.

Significance levels: ^***^ *p*<0.01; ^**^ *p*<0.05; ^*^ *p*<0.1.

## 4 DISCUSSION

Disclosure of one's HIV status can positively impact psychological wellbeing and mental health of PLHIV through a number of social and behavioural pathways, including the receipt of different types of support (social, emotional, material and others), reduced internalized stigma and improved access to care [11, 37–39]. Thus, disclosure of status to someone can be instrumental for the HIV care continuum and health outcomes of pregnant and postpartum WLHIV. Our findings show that about half of the sample of pregnant WLHIV living in Kinshasa had disclosed their status to someone. These observed disclosure rates are lower than pooled estimates of 67% from a systematic review of HIV serostatus disclosure among pregnant and postpartum women in sub‐Saharan Africa [40]. Despite the high (25%) prevalence of IPV reported in the 12 months preceding enrolment, most women who disclosed did so to their partner.

Among women diagnosed prior to the current pregnancy, disclosure of HIV status to male partners was associated with lower levels of current depressive symptoms. This difference persisted even after adjusting for partner's HIV status, experience of IPV, age, number of living children and primigravidae. However, the direction of the association between disclosure and depressive symptomatology was reversed among women who were diagnosed with HIV during the current pregnancy, that is disclosure of HIV status to a partner was associated with more depressive symptoms. Few studies have evaluated disclosure and depression during pregnancy and have reported inconsistent results. For example, a study of 350 WLHIV in South Africa who initiated ART during pregnancy did not find an association between disclosure to a male partner or to family/community member with depressive symptoms during pregnancy [12]. However, in a U.S.‐based study of WLHIV, disclosure of HIV status during pregnancy to the partner or a family member was found to be associated with lower depression symptoms in the postpartum [39].

One hypothesis explaining these results could be that the impact of disclosure on depressive symptoms is mediated by social or emotional support, and that among recently diagnosed women, there has not been enough time to receive such support, regardless of disclosure status. Findings from a study of 244 HIV‐positive and HIV‐negative participants in Uganda that examined disclosure and depressive symptom trajectories may support this hypothesis [12]. The authors reported that HIV status disclosure to a partner on a particular day was associated with higher depressive symptoms that day. Notwithstanding this temporary spike in depressive symptoms, depression scores among those who disclosed to their partner within the first 28 days after diagnosis were significantly lower by the end of the 28 days as well as by 3 and 6 months after diagnosis compared to those who did not disclose [12].

Previous studies have reported higher depression symptoms among women who have disclosed their HIV status during pregnancy, and caution is generally counselled for pregnant women willing to disclose because of risks of negative outcomes, such as IPV or abandonment by a partner [14]. Although 25% of pregnant women in our sample reported experiencing IPV in the past 12 months, the association between disclosure and depressive symptoms was not modified by IPV nor did adjustment for IPV change the effect of disclosure on depression substantially. However, non‐disclosure rates were high even among women who were diagnosed prior to the current pregnancy. This suggests the need to confirm our findings of little effect of IPV on disclosure in another setting or in a prospective study.

Our study should be viewed considering some limitations. Given the cross‐sectional design, we cannot determine the temporality of the association between depressive symptoms and disclosure status. It is possible that women experiencing high level of depressive symptoms chose to disclose their status as a means to alleviate their distress. Longitudinal studies are needed to better characterize the effect of HIV disclosure on depressive symptoms particularly because they may taper off with time regardless of disclosure and/or received social support. While assessing those longitudinal patterns, it is also necessary to account for virological suppression. Although we did not find a statistically significant association between suppressed viral load and depressive scores, high‐level viremia may decrease postpartum among women recently diagnosed for HIV and placed on ART, urging for prospective research to refine observed associations.

We relied on self‐reported measures that could introduce social desirability bias. The questionnaires included sensitive questions and it is possible that women under‐reported depressive symptoms and/or IPV. Depressive symptoms were classified based on symptom criteria and diagnostic interviews were not conducted. Questions could have been misinterpreted or instruments may not match local constructs of disease. We also lacked measures to examine social and emotional support. Finally, despite that all health zones in Kinshasa are represented in our sample, consistent with the Kinshasa Province population distribution, our participants were predominantly urban women and our results may not be generalizable to women living in rural areas.

Despite limitations, this study has several strengths. First, we had a large sample size that allowed us to stratify by time of HIV diagnosis and examine differences in the association between disclosure and depressive symptoms among the two groups of women living with HIV. Second, this study fills a gap in the literature and advances the understanding of disclosure and depressive symptoms during pregnancy among WLHIV.

## 5 CONCLUSIONS

Women in our sample mostly disclosed their HIV status to their partners. Disclosure of HIV status to one's partner was associated with lower depressive symptoms among women who knew their HIV status prior to current pregnancy but not among those who were diagnosed during this pregnancy, suggesting that it might take time for disclosure to produce its effect. Our findings are consistent with cross‐national evidence on the negative association between HIV status disclosure and depressive symptoms in pregnant and postpartum WLHIV. There is a need for prospective research on longitudinal trajectories of disclosure and their association with depressive symptoms.

## COMPETING INTERESTS

The authors have no competing of interests to declare.

## AUTHORS’ CONTRIBUTIONS

MY conceptualized the study and acquired funding. BK, FLK, NR, DK, PB, MY and NZ acquired the data. NZ, IF and MY performed data analysis and drafted the first manuscript version. AA, AMP and KEL discussed the results and contributed to the final manuscript. All authors read and approved the final manuscript.

## FUNDING

This study was supported by the President's Emergency Plan for AIDS Relief (PEPFAR) and the National Institute of Child Health and Human Development (NICHD 1R01H087993). MY and NZ are also partially supported by the following grants from NIH: NIHCD 1R01HD105526 and NIAID U01AI096299.

## Data Availability

Data available on request from the authors.
